# Influence of Autism Traits and Executive Functioning on Quality of Life in Children with an Autism Spectrum Disorder

**DOI:** 10.1007/s10803-015-2438-1

**Published:** 2015-04-03

**Authors:** Marieke de Vries, Hilde Geurts

**Affiliations:** Brain and Cognition, Department of Psychology, University of Amsterdam, Weesperplein 4, 1018 XA Amsterdam, The Netherlands; Dutch Autism and ADHD Research Center, Amsterdam, The Netherlands; Dr. Leo Kannerhuis, Amsterdam, The Netherlands

**Keywords:** Autism, Executive functioning, Quality of life

## Abstract

Children with Autism Spectrum Disorders (ASDs) often experience a low Quality of Life (QoL). We studied if IQ, early language development, current autism traits, and daily Executive Functions (EFs) are related to QoL in children (aged 8–12 years) with ASD (N = 120) and typically developing (TD) children (N = 76). Children with ASD showed a lower QoL than TD children. This lower QoL was related to higher levels of autism traits and EF deficits. Moreover, specific autism traits and EFs were related to specific QoL subdomains. The low QoL and the aggravating effects of autism traits and EF deficits indicate a potential to identify and target such factors in treatment to improve QoL.

## Introduction

Children with autism spectrum disorders (ASD) experience deficits in social interaction, communication, and show stereotyped and repetitive behavior (American Psychiatric Association 2000, 2013). Given the pervasive nature of this neurodevelopmental disorder, children with ASD are challenged in various developmental domains which are of importance for the so called Quality of Life (QoL). QoL is often defined as an individual’s subjective perceptions of both positive and negative dimensions of functioning [functioning itself (e.g., “are you healthy?”), evaluations of functioning (e.g., “do you feel well?”), and personalized evaluations of functioning (e.g., “are you satisfied with your health?”)] on multiple domains (physical, psychological, and social) (WHOQOL Group 1995). QoL is a more subjective appraisal of one’s own well-being, as compared to objectively measureable factors such as health-status or functional impairment (Coghill et al. 2009) and has been studied increasingly in people with ASD in the last decade.

QoL is mostly measured with questionnaires either by self-report, or by parent/caregiver report. Parent- and self-report measures of QoL correlate moderately for both children (6–12 year, *r* = .38; Bastiaansen et al. 2004b), and adolescents (13–18 years, *r* = .39; Clark et al. 2014). However, parents tend to interpret their children’s QoL lower than children interpret their own QoL (Clark et al. 2014). Both children with ASD and their parents report relatively spared school-related QoL, but low social QoL (Clark et al. 2014). Although adolescents with ASD are able to describe themselves, their style is qualitatively different from for example adolescents with an intellectual disability (Lee and Hobson 1998). The adolescents with ASD tend to describe themselves more physically, less in a social context, and have a less well established social self-image (Lee and Hobson 1998). Moreover, both children (Begeer et al. 2008) and adolescents (Scheeren et al. 2010) with ASD present themselves differently than typically developing (TD) children, that is, less strategic and convincing. Hence, especially younger children with ASD may be less able to describe themselves adequately, particularly on social subjects. Parents or primary caregivers interact daily with their child, and have a clear view of their child’s daily functioning and QoL (Coghill et al. 2009). Therefore, parent ratings of QoL appear to be the most reliable QoL measure in relatively young children with ASD.

The reported low QoL of people with ASD (Ikeda et al. 2013) appears to be stable over the life-span (van Heijst and Geurts 2014). As compared to adults who were diagnosed with other psychiatric conditions in childhood, such as Attention Deficit Hyperactivity Disorder (ADHD), disruptive behavior disorders, or affective disorders, adults with ASD show both a lower objective and subjective QoL (Barneveld et al. 2014). Objective QoL measures revealed that adults with ASD were more often single, more often institutionalized, lower educated, had less often paid employment, were more often dependent on social security, and used more anti-psychotics. Subjectively the adults with ASD were less positive about work, education, relationships, and future perspective compared to the adults with other psychiatric conditions. This stable, low QoL in ASD suggests an urgency to study if there are specific factors that influence QoL in ASD. If specific factors can be determined, these factors can serve as potential treatment/intervention targets, which would be an important next step to improve QoL in people with ASD. Therefore, we investigated, besides overall QoL in ASD, whether four specific factors (IQ, autism traits, early language development, and daily executive functioning) were related differently to QoL in ASD than in TD children.

First, children with ASD with a higher IQ function better and have a better prognosis than children with ASD with a lower IQ (Howlin et al. 2004). Adults with ASD with a higher IQ lead a relatively more independent life as they live in group homes less often, and have a daily occupation more often (Billstedt et al. 2011). However, the positive effect of IQ on QoL in ASD appears inconsistent (Chiang and Wineman 2014; Renty and Roeyers 2006; van Heijst and Geurts 2014). There probably is a certain threshold. Children with an IQ above 70 have better outcomes than children with an IQ below 70. For those children with an IQ above 70, outcome is diverse and independent of IQ (Howlin et al. 2004). Hence, having an IQ above 70 appears to be a positive marker, but within the IQ range of 70 and up, IQ does not seem to predict QoL.

Secondly, various studies showed that better language skills in childhood are predictive of adult language skills (Howlin et al. 2004) and of overall outcome (Gillespie-Lynch et al. 2012; Howlin et al. 2004). More specifically, important milestones in language development such as age of the first spoken words, and age of the first spoken sentences, and language use at age five are related to adult outcome (Howlin et al. 2004). Therefore it is hypothesized that language skills will predict QoL (Szatmari et al. 2003).

The third potential factor to influence QoL in people with ASD are autism traits, as (parents of) children with more severe autism traits report lower QoL (Ikeda et al. 2013). However, the exact nature of the connection between autism traits and QoL in children and adolescents with ASD is unclear. Autism traits may influence overall QoL (when controlling for IQ; Kamp-Becker et al. 2010), some aspects of QoL (satisfaction and achievement: Kuhlthau et al. 2013), or only specific autism traits may influence QoL (Tilford et al. 2012). Especially social behavior seems to be predictive of QoL (Chiang and Wineman 2014), and especially children with ASD experience social difficulties. Hence, more pronounced autism traits, including more severe social difficulties, may predict lower QoL.

Last, while a growing body of research focuses on executive functions (EF), the relation between QoL and EF has not yet been studied. This is surprising, as an important cognitive theory postulates that EF is one of the underlying problems in ASD (Russell 1997). Moreover, EF deficits can predict lifelong achievement (Diamond 2013), EF correlates strongly with adaptive behavior in ASD (Gilotty et al. 2002), and adaptive behavior predicts QoL (Chiang and Wineman 2014). EF is also considered to influence school functioning (Diamond 2013), but despite reported EF deficits in ASD, school QoL seems relatively spared (Clark et al. 2014). This can probably be explained by the large individual differences in EF in ASD (Pellicano 2010), and, therefore, we hypothesize that especially those children with EF deficits would show a lower QoL, and possibly also lower school QoL.

Previous studies on QoL in children with ASD included relatively small sample sizes. Those studies that did include a large sample size did not include a control group (Kuhlthau et al. 2010), or studied children without an official ASD diagnosis (Lee et al. 2008). Therefore we studied parent rated QoL in a large sample of children with an official and confirmed ASD diagnosis (N = 120), and a TD control group (N = 76). First we wanted to replicate the finding that QoL is lower in children with ASD compared to TD children. Secondly, we studied if IQ, language, autism traits, and EF influenced QoL, and if these predictors were differently related to QoL in children with and without ASD. Finally, we explored if specific autism traits or EFs influenced certain aspects of QoL. Although IQ is not expected to influence QoL in children with an average or above IQ, we did control for IQ. Later language acquisition, more language difficulties at age five, and more autism traits and EF deficits were expected to predict lower QoL. Similar effects were expected in the ASD and TD group. If predictors would influence QoL differently in the ASD and TD group, this could have important implications for ASD treatment: instead of focusing on functions that are considered to be related to QoL in TD children, those functions that influence QoL in ASD should be targeted.

## Methods

### Participants

Families of children with ASD were approached through mental health care institutions and internet advertisements. From 166 families that initially signed up, 34 decided not to participate, and 132 children were screened for a prior independent clinical ASD diagnosis according to the Diagnostic and Statistical Manual of Mental Disorders IV (DSM-IV: American Psychiatric Association 2000), diagnosed by a multidisciplinary team specialized in ASD. The diagnosis was verified when a score at or above the recommended cutoff for ASD of 57 (raw score, 85th percentile) was reached on the Social Responsiveness Scale parent report (SRS: Constantino et al. 2003; Roeyers et al. 2011), and when cutoffs were reached on at least two out of the three domains on the Autism Diagnostic Interview Schedule-Revised (ADI-R: De Jonge and de Bildt 2007; Lord et al. 1994), and when ASD was recognized before the age of 36 months (Gray et al. 2008).

TD children were approached through various primary schools. Of the 104 children that initially signed up, 22 were not able to participate. Eighty-two TD children were screened for ASD, and were excluded if the score on the SRS was 57 or above. Moreover, both children with ASD, and TD children needed to meet the following inclusion criteria (1) age between 7 and 12 years; (2) IQ above 80 as measured with two subtests of the Dutch version of the Wechsler Intelligence Scale for Children (WISC-III: Bodin et al. 2009; Kort et al. 2002; Sattler 2001), and (3) absence of a seizure disorder.

Twelve children with ASD were excluded after screening (2 ADI-R, 6 IQ, 4 personal circumstances). Forty children with ASD used psychotropic medication [17 abstained during appointments (16 methylphenidate, 1 dexamphetamine), 23 continued (14 methylphenidate, 2 atomoxetine, 5 antipsychotics, and 2 antidepressants)]. Six TD children were excluded after screening (3 IQ, 2 SRS, 1 personal circumstances). Consequently we included 120 children with ASD (years since multidisciplinary clinical diagnosis M = 2.1, SD = 1.7, N = 108; 12 parents did not indicate the date of diagnosis) and 76 TD children for data analyses.

### Measurements

ADI-R: The ADI-R (Lord et al. 1994; Rutter et al. 2003) is an extensive semi-structured interview including three domains of autism symptoms (social, communication, and repetitive behavior). Reaching specified cutoffs on these domains, and clear indications of abnormalities in development before 36 months, suggests a DSM-IV (American Psychiatric Association 2000) or ICD-10 (World Health Organisation 1992) diagnosis of autism (Gray et al. 2008; Rutter et al. 2003). Besides using the ADI-R to verify the ASD diagnosis, we used the ADI-R to assess language development as a predictor. Three language related predictors were included; (1) age in months of the first words; (2) age in months of the first sentences; and (3) a composite of language use at age 4–5 years. A composite score (Howlin et al. 2004) based on the ADI (the precursor of the currently used ADI-R) included utterance length, spontaneous communication, conversational ability, reporting of events, amount of social communication, and intonation and vocal expression. On the ADI-R utterance length, spontaneous communication, and reporting of events are covered in the items 30 (overall level of language), and 41 (current communicative speech), and conversational ability, amount of social communication, and intonation and vocal expression are covered respectively in the items 35 (reciprocal conversation), 34 (social vocalization/“chat”), and 40 (intonation/volume/rhythm/rate). Items were scored as 0: little or no abnormality, 1: mild problems, 2: frequent and sufficient difficulties, or 3: frequent and severe problems. Hence, a higher score indicates less well developed language. We used the scores of functioning at age 4–5 years (raw score, predictor).

SRS: The SRS (Constantino et al. 2003; Dutch version: Roeyers et al. 2011) is a reliable and valid questionnaire measuring autism characteristics (Bölte et al. 2008). The SRS (65 items, 4-point Likert-scale) includes five subscales: Social Awareness (eight items), Social Cognition (12 items), Social Communication (22 items), Social Motivation (11 items) and Autistic preoccupations and Mannerisms (12 items). The SRS was used as a screening to verify ASD, and to measure autism traits (raw score, predictor).

IQ: The WISC-III subtests Vocabulary and Block Design (Kort et al. 2002) correlate highly with Full Scale IQ, have good reliability (Legerstee et al. 2004; Sattler 2001), and were used to estimate IQ (standard score, screening and predictor).

BRIEF: The Behavior Rating Inventory of Executive Function (BRIEF: Gioia et al. 2000; Dutch Version: Smidts and Huizinga 2009) has high to very high internal consistency and test–retest stability (Huizinga and Smidts 2010). The BRIEF (75 items, 3-point Likert-scale) includes 8 subscales; Inhibit (10 items), Shift (8 items), Emotional control (10 items), Initiate (8 items), Working memory (10 items), Plan/organize (12 items), Organization of materials (6 items), Monitor (8 items). The total scale and the subscales were used to measure daily EF (raw scores, predictor).

PedsQL: The Pediatric Quality of Life Inventory (PedsQL: Bastiaansen et al. 2004a; Varni et al. 2001) has satisfactory reliability and validity (Bastiaansen et al. 2004a). The PedsQL (23 items, 5-point Likert scale) includes four subscales; Physical (8 items), Emotional (5 items), Social (5 items), and School (5 items) functioning. The PedsQL is a feasible, reliable, and valid instrument to measure QoL in the general population, as well as clinical groups (Varni et al. 2003). The total scale and the subscales were used to measure QoL (raw score, outcome).

### Procedure

First, written informed consent, SRS, and screening questionnaires were obtained. In the ASD group, during screening the ADI-R was administered to parents, and the WISC-III subtests to the children. In a next appointment the parents filled out the questionnaires.^1^ In the TD group the WISC-III subtests were administered to the children during the screening, and the parents filled out the questionnaires at home. Children received a small gift for participating, and parents of the children with ASD received a report with the ADI-R results, and their travel expenses were largely covered.

### Statistical Analyses

First the ASD and TD group were compared with one way ANOVAs on differences in age, IQ, autism traits, EF, QoL, and subdomains of QoL (Physical, Emotional, Social, and School functioning). Gender was compared with a Chi square test. Second, to study predictors, and to study if predicting values differed between the ASD and TD group, we performed a stepwise regression analysis with QoL as dependent variable and IQ, autism traits, EF, and interaction terms of these predictors with group (ASD/TD) as independent variables. Third, as language measures were not available for the TD group, we repeated this analysis in the ASD group including age of first word, age of first sentence, and language at age 4–5 years as independent variables. Finally, we explored if specific autism traits or specific EFs were related to certain aspects of QoL, as previous studies reported subscale correlations, despite absent total measurement correlations (e.g., Gilotty et al. 2002). Four regression analyses, with each PedsQL subscale as dependent variables, were performed. We checked if the SRS subscales and BRIEF subscales correlated with the PedsQL subscales and the SRS and BRIEF subscales that correlated sufficiently (*r* > .25*, or p* < .10) with a PedsQL subscale were included as predictors. Bonferroni corrections for multiple testing were applied (ANOVAs ASD/TD comparison: 0.05/5 = 0.01, main regression analyses: 0.05/2 = 0.025).

## Results

### Group Differences

The ASD group consisted of more boys than the TD group (*p* < .001). There were no differences in age and IQ (*ps* > .05, see Table 1). On all questionnaires the ASD group showed higher scores, indicating more difficulties (*ps* < .001). Confidence intervals on the PedsQL subscales (Physical 3.7–6.6, Emotional 4.8–6.7, Social 7.1–9.2, and School 3.8–5.5) indicated that children with ASD scored poorest on social QoL, as the score on the social subscale was higher, and the confidence interval did not overlap with the confidence intervals of the other subscales.

**Table 1:** Mean (standard deviations) demographic, predictor, and outcome measures

Measure	Group						Group comparison		*η*_*p*_^*2*^
	TD N = 76			ASD N = 120			*χ* ^2^	*p*	
Gender m/f	43/33			108/12			(1)29.4	<.001	
	N	M(SD)	Range	N	M(SD)	Range	*F*(1194)	*p*	
Age	76	10.1 (1.2)	8–12.9	120	10.2 (1.3)	7.3–12.6	0.0	.86	.00
Intelligence Quotient	76	105.8 (18.4)	81–154	120	110.9 (20.6)	81–170	3.2	.08	.02
Autism traits^a^	76	22.4 (12.8)	3–55	120	96.9 (23.2)	57–152	659.5	<.001	.77
Executive functions^b^	74	104.7 (18.0)	72–162	120	164.9 (18.4)	116–207	497.4	<.001	.72
Age of first word^c^	–	–	–	114	18.5 (7.7)	6–48	–	–	–
Age of first sentence^c^	–	–	–	113	26.1 (9.7)	10–60	–	–	–
Language composite^c^	–	–	–	119	5.4 (2.1)	0–12	–	–	–
Quality of life^d^	73			119			(1190)		
Total		9.2 (7.1)	0–27		32.9 (11.6)	9–65	248.7	<.001	.57
Physical		1.5 (2.4)	0–11		6.6 (5.8)	0–22	51.3	<.001	.21
Emotional		3.5 (2.8)	0–11		9.2 (3.5)	0–20	138.3	<.001	.42
Social		1.7 (2.1)	0–8		9.8 (4.1)	0–19	245.5	<.001	.56
School		2.6 (2.5)	0–10		7.2 (3.3)	0–14	105.3	<.001	.36

In the TD group parents of two children did not return the questionnaires. There were missing values on the PedsQL of one child in the TD group and one child in the ASD group. BRIEF T-scores: TD group M = 41.4, SD = 7.5, range = 26–62, ASD group M = 64.6, SD = 7.4, range = 44–90

^a^Social Responsiveness Scale

^b^Behavior rating inventory of executive function global executive composite raw score

^c^Autism diagnostic interview schedule revised

^d^Pediatric quality of life inventory

### Influence of IQ, Autism Traits and EF on QoL

The model including only EF and autism traits explained the most variance in QoL (*R*^2^ = .70) as both were significantly related to QoL (*ps* < .001, see Table 2). IQ did not seem related to QoL (*p* > .05), therefore IQ was excluded as predictor. Moreover, also none of the interaction terms were significant (*ps* > .05) suggesting that the relation between IQ, autism traits, and EF; and QoL was similar in children with and without ASD. The positive beta-weights (see Table 2) of the remaining predictors indicated that children with more severe EF deficits, and more autism traits, experienced a lower QoL.

**Table 2:** Influence of executive functions and autism traits on quality of life

Predictor variables	Outcome measure			
	Quality of life^a^ (N = 192)			
	*β*	*R* ^*2*^ change	Total *R* ^*2*^	*F*(2189)
			.70***	223.9
Executive functions^b^	.47	.66***		
Autism traits^c^	.40	.05***		

IQ, and predictors*group (autism spectrum disorder vs typically developing) interactions were excluded from the stepwise regression, as these did not seem to influence quality of life. Adding age as a predictor did not change the findings, and age was not significantly related to Quality of life

*** *p* < .001

^a^Pediatric quality of life inventory

^b^Behavior rating inventory of executive function

^c^Social Responsiveness Scale

Given the group difference in gender, we explored if gender influenced the findings. We performed a regression analysis with autism traits, EF, gender, and a group*gender interaction term. Gender, nor the group*gender interaction term predicted QoL (*ps* > .05). Hence, the group difference in gender did not explain the pattern of findings.

### Influence of Language on QoL in the ASD Group

None of the language measures related to QoL in the ASD group (*ps* > .05).

### Exploratory Subscale Predictors

As all subscales of the SRS and BRIEF correlated with the subscales of the PedsQL (*ps* < .05), all were included the regression analyses with respectively the PedsQL subscales Physical, Emotional, Social, and School functioning as dependent variables. The regression analyses revealed that the SRS subscales Social Awareness, and Autistic preoccupations and Mannerisms, and the BRIEF subscales Organization of materials and Monitor were not significantly related to any of the PedsQL subscales. However, the following significant predictors were found (see Table 3): (1) The SRS subscales Social Communication (*p* < .001), and Social Motivation (*p* < .01) were related to the PedsQL subscale Physical functioning. Parents of children with more problems in social communication and motivation, reported lower physical QoL. (2). The SRS subscale Social Motivation (*p* < .01) and the BRIEF subscales Shift (*p* < .001) and Emotional control (*p* < .001) were related to the PedsQL subscale Emotional functioning. Parents of children with more problems in social motivation, poorer cognitive flexibility, and more difficulties in emotional control reported lower emotional QoL. (3) The SRS subscales Social Cognition (*p* < .01), and Social Communication (*p* < .001), and the BRIEF subscales Inhibit (*p* < .001), Shift (*p* < .05), and Initiate (*p* < .01) were related to the PedsQL subscale Social functioning. Parents of children with more communication problems, poorer inhibitory control, poorer cognitive flexibility, and a lower tendency to initiate behavior, reported lower social QoL. Surprisingly, children with poor social cognition showed a higher social QoL. (4) The BRIEF subscales Working memory (*p* < .001), and Plan/organize (*p* < .05) were related to the PedsQL subscale School functioning significantly. Parents of children with more problems in working memory, and planning and organizing skills, reported lower school QoL.

**Table 3:** Influence of autism traits and executive functions on quality of life

Predictor variables	Outcome measures															
	Quality of life: subscales pediatric quality of life inventory															
	Physical (N = 194)				Emotional (N = 194)				Social (N = 193)				School (N = 193)			
	β	*R* ^*2*^ change	Total R^2^	*F*(2191)	β	*R* ^*2*^ change	Total R^2^	*F*(3190)	β	*R* ^*2*^ change	Total R^2^	*F*(5187)	β	*R* ^*2*^ change	Total R^2^	*F*(2190)
			.32***	44.5			.57***	82.8			.69***	81.3			.67***	196.2
*Autism traits* ^a^																
Social cognition									−.33	.01**						
Social communication	.30	.29***							.68	.62***						
Social motivation	.29	.03**			.22	.02**										
*Executive functions* ^b^																
Inhibit									.22	.03***						
Shift					.28	.50***			.17	.01*						
Emotional control					.34	.05***										
Initiate									.16	.01**						
Working memory													.71	.67***		
Plan/organize													.14	.01*		

For each PedsQL subscale a regression analyses was performed. Autism traits (Social Responsiveness Scale subscales) and Executive Functions (Behavior Rating Inventory of Executive Function subscales) that correlated with these PedsQL subscales (*r* > .25*, or p* < .10) were included as predictors

* *p* < .05; ** *p* < .01; *** *p* < .001

^a^Social Responsiveness Scale

^b^Behavior Rating Inventory of Executive Function

## Discussion

In a large sample of children with ASD and a TD control group, we studied QoL and several possible predictors. As expected children with ASD showed a lower QoL in all subdomains (Physical, Emotional, Social, and School) as compared to the TD children. Indeed, children with more autism traits and EF deficits showed a lower QoL. However, in the current study both IQ and language appeared unrelated to QoL. When focusing on specific subdomains of QoL, the current findings suggest children with more problems in social communication and motivation showed lower physical QoL, while children with less social motivation, and poor cognitive flexibility and emotional control showed lower emotional QoL. Also, lower social QoL is reported for children with poor social communication, inhibition, cognitive flexibility, and tendency to initiate behavior, and *better* social cognition. Finally, our exploratory analyses revealed that especially children with poor working memory, planning and organizing skills had a lower school QoL.

Our findings are in line with the consistently reported low QoL, and low social QoL in ASD (Ikeda et al. 2013; van Heijst and Geurts 2014). We did, however, also observe low school QoL, in contrast to previously reported spared school QoL in adolescents with ASD (Clark et al. 2014). Possibly, adolescents with ASD do not experience a low school QoL, while children with ASD do. However, in this previous study ASD was not confirmed with a standardized measure. As our findings indicate that the severity of autism traits relate to QoL, mild autism traits in the sample of Clark et al. (2014) could explain why they reported a relatively good school QoL. Alternatively, the previous study sample (N = 22: Clark et al. 2014) may just have been too small to find differences in the heterogeneous ASD population, although the effect size in the current study was large. For example, working memory and inhibition deficits could be detected in a large group of children with ASD, while only a small subgroup actually showed such deficits, and seemed accountable for the findings (de Vries and Geurts 2014). The quality of the school a child attends may influence school QoL. In the current study, children who attended special education (26 %) did not differ in school QoL (*p* > . 05) from children who attended regular education. However, one might expect that in countries with poorer education systems, children with ASD might experience an even worse school QoL. Hence, although social QoL appears more affected than school QoL, the current findings do indicate that children with ASD have a lower QoL on all subdomains, including school.

In line with previous studies (Chiang and Wineman 2014; Howlin et al. 2004; Renty and Roeyers 2006; van Heijst and Geurts 2014) we did not find that IQ was related to QoL in a sample of children with an average or above IQ. The current findings, therefore, substantiate the idea that if IQ is above the threshold of 70 (Howlin et al. 2004), it does not influence QoL.

Despite previous findings that childhood language predicts adult outcome in ASD (Gillespie-Lynch et al. 2012; Howlin et al. 2004; Szatmari et al. 2003), we did not find a relation between language and QoL in children with ASD. As we only included verbal children with an IQ > 80, the variety in language problems in the current study may have been too small to influence QoL. Although very poor language development seems to predict poor adult outcome, it is less clear if better language development also predicts a better outcome (Howlin et al. 2004), which may also be true for childhood QoL. Children in the current sample showed relatively good language development; 94 % of the children had a good overall level of language at age five, 87 % had said a first word within the normal range (<24 months), and 81 % said a first sentence within the normal range (<33 months). The influence of language development on outcome seems quite specific; it relates to adult social functioning (Gillespie-Lynch et al. 2012), later structural language abilities (Kenworthy et al. 2012), and communication skills (Gillespie-Lynch et al. 2012; Kenworthy et al. 2012; Szatmari et al. 2003), but not to autism symptoms (Kenworthy et al. 2012; Szatmari et al. 2003), such as non-verbal communication and restricted and repetitive behavior (Gillespie-Lynch et al. 2012), nor to adaptive social skills (Kenworthy et al. 2012). The reported (social) communication problems in the current sample may therefore be too limited and subtle to detect a relation with QoL. In sum, in children with ASD with relatively good verbal development and an average or above IQ early language development did not influence childhood QoL.

In line with previous studies (Chiang and Wineman 2014; Ikeda et al. 2013; Kamp-Becker et al. 2010; Kuhlthau et al. 2013; Tilford et al. 2012), we found that autism traits influence QoL. Moreover, we also found that EF was related to QoL. Children with more autism traits, and more EF difficulties, experienced a lower QoL. The finding that EF relates to QoL may have important implications for treatment. Besides focusing on autism traits, targeting EFs may be fruitful in children with ASD. More specifically, as different autism traits and EFs influence different subdomains of QoL, it may be useful to tailor treatment. For example, a social communication training may be particularly helpful for children who experience a low social QoL, while a working memory training may be fruitful for children who experience low school QoL. Although the current findings on subdomains need to be interpreted carefully, they do warrant more thorough research on tailoring treatments for ASD. Although some of the subscale findings seem quite probable (e.g., the relation between less social motivation, poor emotional control, and lower emotional QoL, and the relation between poor working memory, planning and organizing skills, and a lower school QoL), the relation between low social QoL and *better* social cognition might seem somewhat counterintuitive. However, children with better social cognition may have better insight in their social functioning. Children with ASD with lower self-perceived social competence, report more depressive symptoms (Vickerstaff et al. 2007). In line, children who are aware that their social functioning is poor (hence, have better social cognition) may experience a lower social QoL.

Some caveats should be mentioned. Firstly, we studied four important potential predictors of QoL in children with ASD, but other predictors may also influence QoL in ASD, such as broader socio-cultural factors such as ethnicity, religion, and social economic status (SES; Coghill et al. 2009); parent–child interactions (Smith et al. 2014); sleep problems (Delahaye et al. 2014); and comorbidities (Chiang and Wineman 2014). This may be interesting for future research. Secondly, recently a specific QoL measure for ASD was developed, including both ASD specific difficulties and general QoL (Eapen et al. 2014). A specific QoL measurement is useful in clinical practice to measure ASD-specific problems and treatment-outcome. However, when comparing different clinical and non-clinical groups of children, using a generic QoL measurement is more objective (Coghill et al. 2009). Especially when interested in the influence of autism traits on general QoL, a specific ASD-QoL measurement might bias the findings, and a general QoL measurement is more reliable. Thirdly, we used parent measures of all constructs. Although comparing multiple measures from the same informants has the advantage that the measures are cohesive, this might have biased the findings. Parents of children with ASD experience more parental stress than parents of TD children (Hayes and Watson 2013), and parental stress may have influenced the findings. However, when rerunning the analysis in the TD group alone, we still found that EF related to QoL. The influence of the autism traits was, however, no longer significant, which was probably because the TD children showed very little autism traits, as TD children with too many autism traits were excluded from participation. Hence, the effect was still found in parents who are considered to report relatively little parental stress. Nevertheless, parent and child expectations with respect to, for example, social functioning may differ. A child with ASD may prefer few social contacts, which parents may consider unfavorable. Indeed parent–child agreement on QoL is particularly poor on social domains (Eiser and Morse 2001). Including other measures, such as EF tasks, and children’s self-reports, might give more insight, and this is an important next step in future research.

In sum, the current study confirms in a large sample that children with ASD show an overall low QoL. Moreover, children with more autism traits, and more EF deficits, experience a lower QoL. QoL is an important measure of treatment outcome, and the current findings suggest that besides autism traits, it may be fruitful to focus on EF in ASD treatment. There are large individual differences in ASD, and the current findings suggest that specific autism traits, and EFs seem to influence certain subdomains of QoL. Therefore, we argue that personalized treatment programs may be fruitful to improve QoL in children with ASD.

## Footnotes

1 NB the children with ASD took part in a larger intervention study (de Vries et al. 2014), and more tasks and questionnaires were administered to all children, and have been reported in previous studies (de Vries and Geurts 2012, 2014).
